# Prevalence of Post-Traumatic Stress Injuries Among Canadian Public Safety Communicators: A Nationwide Comparative Study: Prévalence des blessures de stress post-traumatique chez les communicateurs en sécurité publique canadiens : étude comparative nationale

**DOI:** 10.1177/07067437261468881

**Published:** 2026-07-18

**Authors:** Steve Geoffrion, Constance Boulé, Filippo Rapisarda, Stephen Czarnuch, Rosemary Ricciardelli

**Affiliations:** 1 5622 School of Psychoeducation, University of Montreal, Montreal, QC, Canada; 2 578596 Centre de Recherche de l'Institut Universitaire en Santé Mentale de Montréal, Montreal, QC, Canada; 3 7512 School of Maritime Studies, Memorial University, St. John's, NL, Canada

**Keywords:** public safety communicators, call-takers, dispatchers, traumatic event exposures, post-traumatic stress injuries, suicide behaviors, comparative study, mental health

## Abstract

**Background:**

Public safety communicators (PSCs, 911 call-takers, dispatchers, and others) play a critical role in Canada's emergency response system, yet they remain largely understudied compared with other public safety professionals (PSP). Although PSCs do not intervene directly at emergency scenes, they are routinely exposed to potentially psychologically traumatic events and high operational stress during calls, placing them at elevated risk for post-traumatic stress injuries (PTSIs). This study aimed to (1) estimate the prevalence of various PTSIs and suicidal behaviors among Canadian PSCs, (2) compare the prevalence with those reported for other Canadian PSP, and (3) identify associated sociodemographic factors.

**Methods:**

The final analytic sample included 508 PSCs. Participants completed validated screening measures assessing symptoms of post-traumatic stress disorder, major depressive disorder, generalized anxiety disorder, panic disorder, and alcohol use disorder, along with questions regarding suicidal behaviors.

**Results:**

Nearly half of PSCs (48.6%) screened positive for at least 1 PTSI, exceeding the prevalence observed among other Canadian PSP. Symptoms of PTSD, depression, anxiety, and panic disorder were significantly higher, while alcohol abuse disorder symptoms were comparable to levels observed among other PSP. Suicide attempts were also significantly more frequent in our sample. Being female emerged as the strongest predictor of screening positive for a PTSI, whereas higher educational attainment was associated with reduced odds of both PTSI symptoms and suicidal ideation.

**Conclusions:**

These findings highlight PSCs as a high-risk and overlooked subgroup within public safety. Trauma-informed training, targeted organizational adjustments, and profession-specific support resources are essential to mitigate the significant psychological burden on this workforce and foster long-term occupational well-being.

## Plain Language Summary Title

Mental health among Canadian public safety communicators: a nationwide comparative study

Plain Language Summary

Why was the study done? Public safety communicators (PSCs), including 911 call-takers and dispatchers, handle emergency calls daily and are exposed to stressful and potentially traumatic situations, but little is known about their mental health.

What did the researchers do? The study surveyed 508 Canadian PSCs about symptoms of stress, depression, anxiety, panic reactions, alcohol use, and suicidal thoughts. The researchers also compared their results with existing research on other public safety workers, including firefighters, police officers, and other PSCs, and examined whether sociodemographic factors predicted mental health outcomes.

What did the researchers find? Almost half of PSCs screened positive for at least one mental health condition. Rates of stress, depression, anxiety, and panic were higher than in other public safety workers. Suicidal thoughts were also more common. Female sex increased risk, while higher education reduced risk.

What do the findings mean? PSCs are at higher risk for mental health difficulties than often recognized. Interventions such as trauma-informed training, workplace support, and profession-specific mental health resources are needed to help protect this workforce. The results also encourage research focused specifically on PSCs, rather than treating them only as a subgroup in broader public safety studies.

## Résumé

**Contexte:**

Les communicateurs de la sécurité publique (CSP: opérateurs 911, répartiteurs, etc.) jouent un rôle essentiel dans le système d’intervention d’urgence du Canada, mais ils demeurent en grande partie sous-étudiés par rapport aux autres professionnels de la sécurité publique (PSP). Bien que les CSP n’interviennent pas directement sur les lieux d’urgence, ils sont régulièrement exposés à des événements potentiellement traumatisants sur le plan psychologique et à un stress opérationnel élevé pendant les appels, ce qui les expose à un risque élevé de blessures de stress post-traumatique (BSPT). Cette étude visait à 1) estimer la prévalence de divers BSPT et comportements suicidaires chez les CSP canadiens, 2) comparer la prévalence avec celles rapportées pour d’autres PSP canadiens et 3) déterminer les facteurs sociodémographiques associés.

**Méthodes:**

L’échantillon analytique final comprenait 508 CSP. Les participants ont répondu à des questionnaires de dépistage validés évaluant évaluant les symptômes du trouble de stress post-traumatique, du trouble dépressif majeur, du trouble anxieux généralisé, du trouble panique et du trouble d’usage de l’alcool, ainsi qu'à des questions concernant les comportements suicidaires.

**Résultats:**

Près de la moitié des CSP (48.6%) ont obtenu un résultat positif pour au moins une BSPT, dépassant la prévalence observée chez les autres PSP canadiens. Les symptômes du TSPT, de la dépression, de l’anxiété et du trouble panique étaient significativement plus élevés, tandis que les symptômes du trouble d’usage de l’alcool étaient comparables aux niveaux observés chez d’autres PSP. Les tentatives de suicide étaient également beaucoup plus fréquentes dans notre échantillon. Le sexe féminin est apparu comme le facteur prédictif le plus important d’un résultat positif au dépistage de BSPT, tandis qu’un niveau de scolarité plus élevé a été associé à une diminution de la probabilité des symptômes de BSPT et des idées suicidaires.

**Conclusions:**

Ces résultats mettent en évidence que les CSP constituent un sous-groupe à risque élevé et négligé au sein de la sécurité publique. La formation sensible aux traumatismes, des ajustements organisationnels ciblés et des ressources de soutien propres à la profession sont essentiels pour atténuer le fardeau psychologique important imposé à ce personnel et favoriser le bien-être professionnel à long terme.

## Introduction

Public safety communicators (PSCs), including 911 operators, call-takers, and dispatchers, serve at the front line of Canada's emergency first response.^1,2^ Their responsibilities include collecting essential information, providing immediate instructions, coordinating emergency services, and offering psychological reassurance to callers. Although they do not intervene directly in the field, PSCs are routinely exposed to potentially psychologically traumatic events (PPTEs) in the course of their duties.^3^ According to the *Diagnostic and Statistical Manual of Mental Disorders* (DSM-5), PPTEs involve exposure to actual or threatened death, serious injury, or sexual violence.^4^ The exposure can occur through both direct and indirect means, including repeated or extreme exposure to details of events experienced by another person. Emergency calls are frequently made in moments of acute emotional distress, during which callers convey vivid descriptions of what they are experiencing. A Canadian study of public safety professionals (PSP) revealed that PSCs experienced at least 15 different types of PPTEs (e.g., assaults and road accidents) in their careers.^5^ An estimated 32% of emergency calls induce clinically significant peritraumatic distress in this workforce, marked by intense emotional responses such as fear, horror, and helplessness.^6^ This is particularly prevalent in cases involving children, domestic violence, suicide, or serious road accidents.^7^

Beyond repeated PPTE exposure, PSCs face a range of operational stressors inherent to their work.^5,8^ The emergencies are unpredictable, yet PSCs are required to strictly follow standardized protocols.^3,7^ They have minimal control over unfolding situations and limited ability to influence outcomes, contributing to a sense of helplessness. These operational stressors are further intensified by excessive workloads and high call volumes, which frequently make it difficult to take adequate breaks or recover between calls.^9^ At the organizational level, PSCs report facing staff shortages, inconsistent leadership, bureaucratic burden, and inadequate supervision and resources, compounded by atypical shift patterns that may contribute to poor lifestyle habits and exacerbate PTSIs.^9,10^ Qualitative evidence further suggests that PSCs often feel invisible and undervalued within their organizations, frequently excluded from post-incident support services and recognition ceremonies available to other first responders,^3,7,11,12^ which discourages help-seeking and further aggravates occupational stress.^10,13^ Taken together, these cumulative demands significantly increase vulnerability to post-traumatic stress injuries (PTSIs).^14,15^

PTSI is a non-clinical term encompassing a range of mental health challenges, including post-traumatic stress disorder (PTSD), anxiety disorders, mood disorders, substance use disorders, and suicidal behavior.^16^ PTSI refers to symptoms resulting directly from the work demands and conditions. In a nationwide study by Carleton et al.,^17^ 48.4% of PSCs met the clinical threshold for at least 1 PTSI. The most prevalent were major depressive disorder (MDD, 33.2%), (PTSD, 18.3%), generalized anxiety disorder (GAD, 18.0%), social anxiety disorder (SAD, 16.9%), and alcohol use disorder (AUD, 7.2%). Additionally, 9.5% reported experiencing suicidal ideation within the past year, with 2.5% having formulated a suicide plan and 0.4% having attempted suicide.^18^ PTSIs can affect task performance and mental health, contributing to higher absenteeism and leave, reduced job performance, and increased turnover intentions.^19–21^

PSCs remain significantly understudied compared with firefighters, police officers, and paramedics, despite their vital role in public safety.^14^ For instance, in Carleton et al.'s^17^ large-scale study among public safety personnel, approximately 4% of participants were PSCs (as inferred from reported data), illustrating how research in this area remains limited. This knowledge gap limits the development and effective implementation of evidence-based interventions tailored to the needs of PSCs managing PTSIs. Consequently, it affects their well-being and the quality of services provided to the public. Thus, the current study aims to: (1) estimate the prevalence of PTSIs among Canadian PSCs, (2) compare these findings with existing Canadian data at the broader PSP level, with a complementary comparison among a PSC-specific subsample, and (3) identify key sociodemographic factors associated with the presence of PTSIs.

## Methods

### Participants

Inclusion criteria for the present study included employment in Canada as a PSC for 911, police, fire, or ambulance services. Eligible participants held roles such as call-taker, dispatcher, manager, or quality assurance/quality improvement supervisor or trainer. They worked within a public safety answering point or a contracted communication service, such as OnStar or a VoIP-based provider. Most participants were employed full- or part-time (92.7%), with smaller proportions on leave (3.2%) or students (1.0%); 3.1% did not disclose their employment status. Regarding years in their current role, most participants reported 10 or more years of experience (33.9%), followed by 5 to 10 years (23.3%), 2 to 5 years (21.9%), and 2 years or less (20.9%). Overall, 696 PSCs completed the survey. We excluded 176 respondents with missing data in more than half of the sociodemographic or PTSI questionnaires; the final analytic sample comprised 508 participants. This approach ensured a missing rate below 5% for sociodemographic variables and enabled the computation of combinations involving 3 or more mental disorders, facilitating comparison with existing literature.

### Procedure

Potential participants were initially notified about the study through a recruitment email distributed to the entire membership of the Association of Public-Safety Communications Officials (APCO Canada). Additional participants were recruited through direct online searches for public safety answering points, outreach to emergency communication oversight organizations, and exploration of social media platforms such as Facebook and X. To protect the confidentiality of participating services and individuals, organizational affiliations are not disclosed. The recruitment email outlined the survey purpose, emphasized its anonymous nature, and invited interested PSCs to voluntarily access the online informed consent page via the provided link. After consenting to participate, PSCs were guided through the process of creating a unique, private access code. This code enabled participants to complete the questionnaire over multiple sessions without generating duplicate entries, while preserving their anonymity. Data collection occurred between November 2020 and April 2021. The survey was delivered through an online questionnaire hosted on the Qualtrics platform provided by Memorial University and received ethical approval from the Newfoundland and Labrador Human Research Ethics Board (File #20210168; Approval date October 13, 2020) prior to data collection commencing.

### Measures

A cross-sectional survey was developed to capture a statistically representative snapshot of self-reported PTSIs among Canadian PSCs. The survey design used measures consistent with those used by Carleton et al.^17,18^ enabling comparisons with the broader PSP population (e.g., police officers, firefighters, paramedics) as well as with their PSC-specific subsample. The use of identical measures ensures validity across comparisons, without requiring demographic matching given the distinct occupational and demographic profile of PSCs relative to other PSP categories. PTSIs were assessed through self-report screening measures administered in the survey. These included the PTSD checklist for DSM-5 (PCL-5)^22^ to measure PTSD symptoms, the 9-item patient health questionnaire (PHQ-9)^23^ for MDD measures, the panic disorder severity scale–self-report (PDSS-SR)^24^ to assess panic disorder, the 7-item GAD scale (GAD-7)^25^ for measuring GAD, and the AUDs identification test (AUDIT)^26^ for assessing AUD. Participants completed each instrument according to its standardized reference period: past month for the PCL-5, past 14 days for the PHQ-9 and GAD-7, past 7 days for the PDSS-SR, and past year for the AUDIT.

Closed-ended (yes/no) questions, consistent with those used by Carleton et al.,^18^ were used to assess suicide behaviors within the past 12 months. Suicidal ideation was evaluated using the question “Have you ever seriously contemplated suicide?”; suicide planning was assessed with “Have you ever made a plan to attempt suicide?”, and suicide attempts were assessed with “Have you ever attempted suicide?” For each item, if participants responded “yes,” a follow-up question asked, “Has this occurred within the past 12 months?” Sociodemographic information was also collected within the online survey. Participants were asked to report their sex (male, female, or rather not say/other) and gender identity (man, woman, transgender or other, or rather not say). Age was recorded in categorical ranges (19–29, 30–39, 40–49, 50–59, and 60+ years). Educational attainment was classified as high school graduate or less, some postsecondary education, or university degree/4-year college or higher. Marital status was assessed using the following categories: single, separated/divorced, married/common-law, remarried, widowed, or other.

### Analyses

Descriptive statistics were computed to summarize participants’ sociodemographic characteristics and symptom measures. For each mental health screening instrument, established cutoff scores determined the presence of clinically significant symptoms. Prevalence estimates for each disorder and aggregated symptom categories were calculated and compared with previously published data on Canadian PSP using chi-square (*χ*^2^) tests, including a primary comparison with the broader PSP population and a complementary comparison with a PSC-specific subsample from Carleton et al.^17,18^ To examine the sociodemographic determinants of clinical mental health outcomes, binary logistic regression analyses were conducted. Two separate models were estimated: one predicting the likelihood of screening positive for one or more mental health conditions, and one predicting the presence of suicidal ideation within the past 12 months. Data were analyzed using R statistical software version 4.1.1.^27^

## Results

Table 1 describes participants’ sociodemographic characteristics. Most participants indicated being female (78.4%), identified themselves as woman (77.8%), and were between 30 and 49 years old. In terms of educational attainment, 58.6% reported having some post-secondary education, while 27.9% held a university degree or higher. Most respondents were married or in a common-law relationship (61.6%), followed by single individuals (23.1%). Table 2 summarizes the descriptive statistics for the PTSI-related measures.

**Table 1.** Sample Descriptives.

Sex	
Male	21.1% (107)
Female	78.1% (397)
Rather not to say or other	0.6 (3)
Missing	0.1% (1)
Gender	
Man	21.1% (107)
Woman	78.0% (396)
Rather not to say or other	0.6% (5)
Missing	0 (0)
Age	
19–29	15.7% (80)
30–39	35.2% (179)
40–49	28.9% (147)
50–59	15.4% (78)
60+	3.7% (22)
Education	
High school graduate or less	13.0% (66)
Some post-secondary school	55.3% (281)
University degree/4-year college or higher	27.9% (178)
Missing	3.7 (19)
Marital Status	
Single	22.6% (115)
Separated/divorced	8.9% (45)
Married/common law	61.6% (313)
Remarried	2.8% (14)
Widowed	1.4% (7)
Missing	4.2% (21)

**Table 2.** Symptoms Scales Mean Scores.

	Mean (SD)
Post-traumatic stress disorder (PCL-5)	22.8 (19.2)
Major depressive disorder (PHQ-9)	8.3 (6.5)
Generalized anxiety disorder (GAD-7)	7.2 (5.8)
Panic disorder (PDSS-SR)	3.9 (5.4)
Alcohol use disorder (AUDIT)	5.4 (4.6)

Table 3 presents the prevalence estimates of clinical PSTI symptoms within the sample and compares these rates with previously published Canadian PSP data, including a complementary PSC-specific comparison. Among participants, 29.4% screened positive for PTSD, a significantly higher proportion than the 23.3% reported in prior PSP studies (*χ*^2^ = 15.3, *p* < .001) and among prior PSC data (18.3%; *χ*^2^ = 10.5, *p* = .001). Major depressive disorder was identified in 35.6% of the current sample, which was significantly higher than the broader PSP estimate (26.4%; *χ*^2^ = 20.0, *p* < .001), but comparable to prior PSC data (33.2%; *χ*^2^ = 0.5, *p* = ns). Clinical symptoms of generalized anxiety were present in 27.8% of participants, exceeding both the broader PSP estimate (18.6%; *χ*^2^ = 23.3, *p* < .001) and prior PSC data (18.0%; *χ*^2^ = 6.4, *p* = .011). Panic disorder was also more prevalent in the current sample (21.9%) than in both the broader PSP data (8.9%; *χ*^2^ = 79.5, *p* < .001) and prior PSC data (7.6%; *χ*^2^ = 17.7, *p* < .001). In contrast, AUD was detected in 5.2% of participants, a rate comparable to both previous PSP data (5.9%; *χ*^2^ = 0.3, *p* = ns) and prior PSC data (7.2%; *χ*^2^ = 2.7, *p* = ns).

**Table 3.** Estimates of PTSI Symptoms and Comparison with Previous Data on PSP.

	PSCs Sample *N* = 508	Comparison Versus Broader PSP Data^17,18^ *N* = 5813		Comparison Versus PSCs Subsample Data^17^	
	% (*n*)	%	*χ*^2^, *p*	%	*χ*^2^, *p*
Recent clinical PTSI symptoms					
PTSD (PCL-5)	29.4% (146)	23.2	15.3***	18.3	10.5**
Major depressive disorder (PHQ-9)	35.6% (181)	26.4	20.0***	33.2	0.5^ns^
Generalized anxiety disorder (GAD-7)	27.8% (133)	18.6	23.3***	18.0	6.4*
Panic disorder (PDSS-SR)	21.9% (100)	8.9	79.5***	7.6	17.7***
Alcohol use disorder (AUDIT)	5.2% (22)	5.9	00.3^ns^	7.2	2.7^ns^
Aggregated estimation					
Any positive screen for an anxiety disorder	30.9% (157)	30.3	2.9^ns^	32.2	0.1^ns^
Any positive screen for a mood disorder	35.6% (181)	29.0	1.1^ns^	36.1	0^ns^
Any positive screen for any mental disorder	48.6% (247)	44.5	8.6 **	48.4	0^ns^
Total number of positive screens					
0 (absence of above threshold symptoms)	51.4% (261)	58.2	21.0***	55.7	1.3^ns^
1	14.0% (71)	15.1		13.9	
2	14.2% (72)	8.7		12.4	
3 or more	20.5% (104)	18.0		17.9	
Suicide behaviors in the last 12 months					
Suicide ideation	7.8% (39)	10.1	2.5^ns^	—	—
Suicide planning	3.3% (17)	4.1	0.5^ns^	—	—
Suicide attempt	0.6% (3)	0.3	9.6 *	—	—

*Note.* Clinical cutoff scores indicating probable disorder: PCL-5 ≥ 33; PHQ-9 ≥ 10; GAD-7 ≥ 10; PDSS-SR ≥ 9; AUDIT ≥ 8. PSP = public safety personnel; AUDIT = alcohol use disorders identification test; GAD-7 = generalized anxiety disorder scale; PCL-5 = posttraumatic stress disorder checklist for DSM-5; PDSS-SR = panic disorder symptoms severity scale, self-report; PHQ-9 = patient health questionnaire.

*p < .05; **p < .01; ***p < .001.

Aggregated estimates revealed that 30.9% of participants screened positive for at least 1 anxiety disorder, a prevalence consistent with both previous PSP data (30.3%; *χ*^2^ = 2.9, *p* = ns) and prior PSC data (32.2%; *χ*^2^ = 0.1, *p* = ns). Similarly, 35.6% screened positive for a mood disorder, showing no significant difference from prior PSP data (*χ*^2^ = 1.1, *p* = ns). The overall prevalence of screening positive for any mental disorder was 48.6%, which was significantly higher than the broader PSP estimate (44.5%; *χ*^2^ = 8.6, *p* = .004), but virtually identical to prior PSC data (48.4%; *χ*^2^ = 0.0, *p* = ns).

The distribution of the number of positive screens in the current sample was as follows: 51.4% (*n* = 261) of participants reported no above-threshold symptoms, 14.0% (*n* = 71) met criteria for 1 condition, 14.2% (*n* = 72) for 2 conditions, and 20.5% (*n* = 104) for 3 or more conditions. In the broader PSP comparison sample, the corresponding rates were 58.2%, 15.1%, 8.7%, and 18.0%, respectively, with a statistically significant difference in the overall distribution (*χ*^2^ = 21.0, *p* < .001). In prior PSC data, the corresponding rates were 55.7%, 13.9%, 12.4%, and 17.9%, respectively; this distribution did not differ significantly from the current PSC sample (*χ*^2^ = 1.3, *p* = ns).

Regarding suicide-related symptoms, 7.8% of participants reported suicidal ideation in the past 12 months, a prevalence not statistically different from previously reported data among Canadian PSP (10.1%; *χ*^2^ = 2.5, *p* = ns). Suicide planning during the same period was reported by 3.3% of the sample and did not differ significantly from prior PSP data (4.1%; *χ*^2^ = 0.5, *p* = ns). Suicide attempts in the past 12 months were reported by 0.6% of participants, a rate significantly higher than previously documented among PSP (0.3%; *χ*^2^ = 9.6, *p* = .022). Comparable suicide-related estimates were not available in the prior PSC comparison data.

### Determinants of Clinical PTSI Symptoms

A binary logistic regression was conducted to examine associations between sociodemographic factors and the likelihood of reporting 1 or more mental-health conditions or suicide planning in the last 12 months (see Table 4). The model that predicted mental health conditions was statistically significant, *χ*^2^ (10) = 23.74, *p* = .009, indicating improved fit over the null model (AIC = 696.56). Sex and education level were significant predictors. Males had lower odds of meeting the cutoff for at least 1 mental-health condition compared with females (OR = 0.45, 95% CI [0.28, 0.70], *p* < .001). Participants with a university degree also showed reduced odds relative to those with high-school education (OR = 0.40, 95% CI [0.21, 0.74], *p* = .004). No significant effects were observed for age or marital status.

**Table 4.** Determinants of Clinical PTSI Symptoms and Suicide Ideation.

	Any Clinical PTSI Symptoms				Suicidal Ideations			
Predictor	*β* (SE)	Wald z	*p*	OR (95%CI)	*β* (SE)	Wald z	*p*	OR (95%CI)
Intercept	0.55 (0.31)	1.74	.082	1.73 (0.94–3.23)	–1.85 (0.52)	–3.60	<.001	0.16 (0.05–0.41)
Sex (ref. female)								
Male	–0.81 (0.23)	–3.48	<.001***	0.45 (0.28–0.70)	–0.21 (0.44)	–0.48	.634	0.81 (0.31–1.83)
Age (ref: 30–39)								
19–29	0.21 (0.29)	0.73	.468	1.23 (0.70–2.16)	0.72 (0.47)	1.51	.130	2.05 (0.79–5.18)
40–49	0.14 (0.23)	0.61	.544	1.15 (0.73–1.81)	–0.02 (0.43)	–0.05	.958	0.98 (0.42–2.27)
50–59	0.27 (0.29)	0.95	.341	1.31 (0.75–2.31)	–1.31 (0.80)	–1.64	.100	0.27 (0.04–1.06)
60+	0.04 (0.48)	0.08	.936	1.04 (0.40–2.72)	–0.86 (1.10)	–0.78	.438	0.43 (0.02–2.55)
Education (ref: high school)								
Post-secondary	–0.52 (0.29)	–1.76	.079	0.6 (0.33–1.05)	–0.68 (0.48)	–1.41	.159	0.51 (0.20–1.39)
University	–0.92 (0.32)	–2.90	.004**	0.4 (0.21–0.74)	–1.10 (0.56)	–1.98	.048	0.33 (0.11–1.02)
Marital status (ref: married)								
Single	0.10 (0.23)	0.44	.658	1.11 (0.70–1.76)	0.06 (0.43)	0.13	.894	1.06 (0.44–2.40)
Separated/divorced	–0.10 (0.33)	–0.30	.761	0.9 (0.47–1.73)	0.97 (0.51)	1.88	.060	2.63 (0.89–6.92)
Other	0.14 (0.38)	0.37	.710	1.15 (0.55–2.42)	–0.74 (1.05)	–0.70	.481	0.48 (0.03–2.47)

*p < .05; **p < .01; **p < .001.

Instead, the model for predicting suicidal ideation was not statistically significant, *χ*^2^ (10) = 14.06, *p* = .17 (AIC = 277.46). Most sociodemographic variables were not significant predictors of suicidal ideation, except for education. Participants with a university degree had lower odds of reporting suicidal ideation compared with those with a high-school education. (OR = 0.33, 95% CI [0.11, 1.02], *p* = .048). Conversely, those who were separated or divorced showed higher odds compared with married/common-law participants (OR = 2.63, 95% CI [0.89, 6.92], *p* = .06). No significant effects were observed for sex, age, or other variables.

## Discussion

Our study aimed to estimate the prevalence of PTSIs and suicidal behaviors among Canadian PSCs and compare these findings with those reported for other Canadian PSPs. Associated sociodemographic factors were also examined. A cross-sectional survey captured a representative snapshot of self-reported PTSIs among PSCs, using a measure to allow comparison with a pan-Canadian study^17,18^ of prevalence among other PSP, including comparisons within their PSC-specific subsample.

### PTSIs and Suicidal Behaviors Among Canadian PSCs

Nearly half of the participants (48.6%) screened positive for at least 1 PTSI, underscoring the substantial psychological burden facing Canadian PSCs. Prevalence of most PTSIs was markedly higher than those reported in the broader PSP population,^17^ with the exception of AUD symptoms, which were comparable. Complementary comparisons with Carleton's PSC subsample^17^ revealed that despite broadly similar mean scores, cutoff-based analyses showed significantly higher prevalence of probable PTSD, GAD, and panic disorder in our sample, suggesting that average symptom levels may not fully capture clinically significant burden at the individual level. This pattern likely reflects differences in the underlying distribution of symptom severity, with a greater proportion of respondents in our study scoring above clinically relevant thresholds. The larger size and dedicated nature of our sample may have increased sensitivity to detect such variations, enabling a more comprehensive characterization of symptom severity among Canadian PSCs. Together, these findings indicate that PSCs may constitute an under-recognized high-risk subgroup for compromised mental health and highlight the importance of PSC-specific research.

The distinct mental health profile observed in our sample may be partly explained by several operational features that differ between PSC work and other PSP roles.^9,10,28^ Although PSCs do not intervene directly at emergency scenes—they are “before the frontline”^12^—the nature of their PPTE exposure may generate distinct stress burdens. Repeated unseen but heard exposure to PPTEs or human suffering can affect individuals’ mental health, particularly when lacking the agency or ability to physically intervene, which often manifests as vicarious traumatization or secondary traumatic stress.^29–31^ Unlike the in-person frontline, PSCs are physically removed from incidents and rely exclusively on verbal communication and remote guidance. This operational context exposes them to acute suffering in real time while being unable to act directly in helping, often eliciting profound feelings of helplessness and moral distress.^7,9^

In addition, chronic exposure to situations in which one feels powerless can undermine a sense of professional efficacy and contribute to PTSI symptoms, particularly of anxiety, depression, and PTSD.^32^ Continuous transitions between emergency calls, without sufficient opportunities for decompression, create uninterrupted stress cycles rarely experienced by field PSP.^9^ Collectively, the operational characteristics of PSC work and the moral challenges inherent to their role likely increase vulnerability to PTSIs.

Findings show significantly higher suicide attempts among Canadian PSCs compared with other PSP, although other suicidal behaviors do not differ. Elevated attempt rates despite similar levels of suicidal ideation may reflect differences in how occupational stress is experienced and processed. Cumulative exposure to PPTEs can impair cognitive flexibility, working memory, and executive function, increasing reliance on maladaptive coping strategies.^33,34^ While all PSP encounter PPTEs, PSCs are uniquely positioned as first-line responders through indirect exposure, often unable to act or resolve situations, which can heighten helplessness and emotional burden.^9^ Compared with field PSP, PSCs have fewer opportunities for immediate problem-solving and peer support, possibly increasing reliance on avoidance or emotional suppression for coping. Over time, even minor stressors may become overwhelming, potentially accelerating the transition from suicidal ideation to attempt. This aligns with models of cumulative stress and allostatic load, where repeated exposure reduces resilience and heightens vulnerability to adverse mental health outcomes.^33–35^

### Associated Sociodemographic Factors

Findings identify biological sex, specifically being female, as the strongest predictor of PTSIs among participants. This pattern aligns with extensive evidence demonstrating that females generally exhibit higher prevalence of PTSD, anxiety disorders, and depressive symptoms than males.^36,37^ Several mechanisms account for these sex-related disparities, including hormonal and neurobiological differences in stress responsivity and heightened vulnerability to vicarious trauma.^37,38^ The PSC workforce includes a higher proportion of women compared with other public safety sectors such as firefighting and law enforcement. In our sample, 78.1% of participants were female, reflecting a profession-specific contextual factor that should be explicitly considered when interpreting PTSI risk profiles for this occupational group, highlighting the need to study PSP professions independently.

Participants with a university degree had lower odds of reporting clinical PTSI symptoms and suicidal ideation compared with those whose highest education was high school, suggesting higher educational attainment may be protective. Higher education is often associated with greater cognitive and coping resources, as well as higher mental health literacy.^39–41^ These capacities may enhance individuals’ ability to manage stress and access social and practical support resources, potentially buffering the impact of repeated occupational stressors.

### Implications for Targeted Intervention, Training, and Prevention

The elevated prevalence of PTSI symptoms and suicide attempts among PSC highlights the urgent need for interventions tailored to this high-risk group. Early detection through routine screening with direct avenues to intervention is critical.^42^ Such measures can enable timely prevention and reduce adverse outcomes. PSC-specific protocols are essential for effectively managing occupational stressors. Multi-session trauma-sensitive training should be integrated into PSCs training early in their career and reinforced continuously to strengthen adaptive coping, resilience, and emotional regulation skills.^43–45^ Such training can also promote early recognition of PTSI symptoms, potentially reducing chronicity and encouraging timely help-seeking. Given the well-documented organizational challenges faced by PSCs,^9,10,13^ adjustments at this level, including optimized shift patterns and access to mental health services familiar with communication center stressors, may represent plausible organizational strategies to reduce workplace vulnerability to PTSIs. Although multiple interventions aim to improve broader PSP mental health, evidence regarding their effectiveness is sparse, particularly within PSC-specific samples,^46,47^ underscoring the need for targeted research in this understudied group. Comparative evidence between organizations that implement these measures and those that do not is currently lacking.

Beyond task-related changes, fostering a supportive work culture in which PPTE exposure is acknowledged and open communication regarding mental health is actively supported is essential for sustaining PSCs’ psychological safety at work.^48,49^ Integrating demographic risk factors, operational demands, and organizational characteristics appears crucial for developing evidence-informed, PSC-specific programs.^50^ Such targeted approaches are likely more effective than broad PSP-wide strategies in promoting long-term resilience and functional performance.

## Directions for Future Research

Future research should examine the longitudinal evolution of PTSIs among PSCs, ideally beginning with mental health baselines established at employee onboarding. Establishing such baselines would enable researchers to distinguish pre-existing conditions from occupationally induced symptoms and more clearly attribute changes in symptom trajectories to the specific demands and exposures associated with PSC-specific demands. Given the distinct occupational realities of PSC, future studies should prioritize dedicated PSC-specific samples rather than subgroups embedded within broader PSP research, as such approaches may obscure clinically significant variations unique to this population. Future studies should also prioritize the use of structured clinical interviews (e.g., the Mini-International Neuropsychiatric Interview [MINI] or the Structured Clinical Interview for DSM-5 Disorders [SCID-5]) alongside self-report measures to support formal diagnostic assessments. Additionally, investigating call content, including child-related incidents, suicides, and violent crimes, may help clarify which exposures most strongly predict PTSIs and thereby inform preventive strategies. Although we caution that all future research should be contextualized, for instance, by recognizing the implications of the sworn/civilian divide on civilians such as PSCs.^51^ Further examination of the interaction between operational stressors and organizational climate factors, including leadership, support, and perceptions of fairness, could inform the development of tailored training programs and interventions, which should subsequently be evaluated using controlled study designs.

## Limitations

This study presents several limitations that warrant consideration. First, the use of a convenience sample, combined with voluntary participation, raises the possibility of selection bias. PSCs who chose to participate may have been particularly willing to discuss mental health or more open to the study topic. Second, all measures relied on self-report questionnaires, introducing potential measurement biases such as social desirability and under- or over-reporting of symptoms. Third, data collection occurred during the COVID-19 pandemic, a period marked by increased stress and strained operational contexts across public safety sectors. Symptom rates may have been amplified or reduced by the broader societal and occupational crisis context. Fourth, it was not possible to verify participants’ employing organizations, preventing us from examining regional or organizational differences in mental health outcomes. Finally, no detailed information was collected regarding participants’ specific job functions (e.g., call taker vs. dispatcher), which is a relevant limitation given that exposure profiles, operational stressors, and emotional demands may vary substantially across roles within PSC work.
